# Cutaneous *Lagenidium deciduum* infection in a patient with relapsed acute myeloid leukemia

**DOI:** 10.1186/s12879-024-09281-5

**Published:** 2024-05-23

**Authors:** Joanna Theophilopoulos, Rebekah King, Autumn Citta, Constance Alford, Natalie Dotson, Connie Cañete-Gibas, Carmita Sanders, Nathan Wiederhold, John A. Ligon, Connie Trieu

**Affiliations:** 1 University of Florida College of Medicine, Gainesville, FL USA; 2 Department of Pediatrics, University of Florida College of Medicine, Gainesville, FL USA; 3 Department of Pediatrics, Division of Pediatric Hematology Oncology, University of Florida College of Medicine, Gainesville, FL USA; 4 Fungus Testing Laboratory, Department of Pathology and Laboratory Medicine, University of Texas Health San Antonio, San Antonio, TX USA; 5 Department of Pediatrics, Division of Pediatric Infectious Diseases, University of Florida College of Medicine, Gainesville, FL USA

**Keywords:** *Lagenidium deciduum*, Lagenidiosis, Oomycosis

## Abstract

**Background:**

*Lagenidium deciduum* is an oomycete that can cause infections in mammals that present similarly to pythiosis and mucormycosis. Most of the existing case reports have occurred in canines and have been fatal. In animals, medical therapy has not been successful, so surgical excision is the mainstay of treatment. *Lagenidium* sp. infections in humans are rare. There is only one case of a human *Lagenidium* sp. infection in the literature, and it presented as an ocular infection. The human ocular infection was resistant to medical therapy and required a penetrating keratoplasty for cure. Additional reports of effective therapy are needed to guide management of this emerging pathogen. We present the first case of a cutaneous *Lagenidium deciduum* infection in a human patient, which is also the first documented case of a *Lagenidium deciduum* infection in an immunocompromised host of any species.

**Case presentation:**

An 18-year-old female with relapsed acute myeloid leukemia, awaiting a haploidentical stem cell transplant, presented with erythematous cutaneous lesions on her left hip and bilateral buttocks that enlarged and blackened over several days. About 1 week later, boil-like lesions appeared on her bilateral buttocks. The skin lesions were initially presumed to be bacterial in origin, so the patient was treated with clindamycin and cefepime with little improvement. Upon further investigation, fungal cultures and skin biopsies revealed aseptate hyphae, so the patient was switched to isavuconazole and amphotericin B due to concern for mucormycosis. Phenotypic characterization and DNA sequencing were performed by the Fungus Testing Laboratory, University of Texas Health Science Center at San Antonio, which identified the causal fungal organism as *Lagenidium deciduum.* All of her cutaneous lesions were surgically excised, and the patient was treated with micafungin, terbinafine, doxycycline, and azithromycin. Micafungin and terbinafine were continued until she achieved engraftment post-transplant.

**Conclusions:**

We report the first successful treatment of a human *Lagenidium* infection in an immunocompromised host through a combination of aggressive surgical excision and prolonged antifungal therapy during the prolonged neutropenia associated with allogeneic stem cell transplant. Prompt diagnosis and management may prevent disseminated oomycosis.

## Background

*Lagenidium deciduum* is a water mold in the class of oomycetes. Other organisms in this class include, but are not limited to, *Pythium insidiosum, Lagenidium albertoi, Lagenidium giganteum*, *Lagenidium juracyae*, *Paralagenidium ajellopsis* and *Paralagenidium karlingii.* On histopathology, *Lagenidium* species (spp.) appear as broad, sparsely septate hyphae with irregular branching [1]. *Lagenidium* infections are commonly compared to pythiosis and mucormycosis because they often have similar clinical presentations and histopathologic features. The clinical presentation of *Lagenidium* infections was first described in a 2003 case series of six dogs from the southern United States, and the term “lagenidiosis” was coined [2]. In the case series, five dogs presented one to 3 months after the development of cutaneous and subcutaneous lesions, and one dog presented with mandibular lymphadenopathy. The cutaneous lesions were described as ulcerated and necrotic, with draining sinus tracts.

*Lagenidium* infection in humans is exceedingly rare. Therefore, approaches to management are extrapolated from cases of human pythiosis. Pythiosis is caused by the oomycetes *Pythium* spp.*,* including *P. insidiosum*, which is the primary species associated with human infections, and *P. aphanidermatum,*which has also been documented to cause disease in humans [3, 4]. It typically presents as vascular or ocular pythiosis but rarely can manifest as cutaneous or disseminated disease. Like *Lagenidium*, *Pythium* has a predilection for invading blood vessel walls. The skin is thought to be the port of entry for vascular pythiosis. Patients typically present with chronic, non-healing skin lesions, arterial insufficiency syndrome, and aneurysms [3]. The mainstay of management for pythiosis is surgical intervention, as the organism is often resistant to medical treatment.

Prior to this report, there was only one case of a human infection with *Lagenidium* sp. reported in the literature, and it presented as severe keratitis in an adult patient in Thailand [5]. Although there are no known cases of *Lagenidium* infections in immunocompromised patients, there are documented cases of pythiosis in neutropenic patients. These case reports have shown that neutropenic patients may present more abruptly and progress to vascular disease more quickly (within a few days to weeks) compared to immunocompetent hosts [3, 6, 7]. In this report, we present the first case of a cutaneous *L. deciduum* infection in a human patient. This is also the first documented case of a *L. deciduum* infection in an immunocompromised host of any species. This case report also highlights the challenges associated with treatment of lagenidiosis in humans.

## Case presentation

An 18-year-old female with relapsed high-risk acute myeloid leukemia (AML) was evaluated for a haploidentical stem cell transplant (HSCT). She was initially diagnosed with high-risk AML in May 2021 (with central nervous system involvement at the time of diagnosis) and completed treatment under the Children’s Oncology Group study protocol AAML0531 in December 2021. In April 2022, she presented with thrombocytopenia and easy bruising, and subsequent bone marrow evaluation revealed relapse of her AML. She completed 1 cycle of relapse therapy with azacitidine, venetoclax and gemtuzumab in early June 2022. Soon after, she developed cellulitis of her right superior-medial gluteal region. She was admitted to a local hospital and initiated on cefepime and vancomycin. Vancomycin was subsequently switched to clindamycin, and a targeted ultrasound of the affected area revealed a 1.2 cm fluid collection with a fistula tracking to the skin. The patient was discharged home on oral clindamycin, and her cellulitis and abscess resolved after several days.

In late June 2022, she developed a new skin lesion on her left hip. The lesion started as a “red spot”, which appeared like “bleeding under the skin.” Over the course of a few days, the lesion became swollen and turned black. One week later, the patient noticed several new lesions on her buttocks around the intergluteal cleft, which she described as “boils”. The patient was then admitted in early July 2022 for further evaluation of her skin lesions.

Of note, the patient reported that she swam in a freshwater river and a chlorinated swimming pool in late May 2022. She also reported swimming in a freshwater lake in late June 2022 (after the development of the lesion on her left hip). She denied any exposure to or recent history of hot tubs, camping, travelling, or methicillin-resistant *Staphylococcus aureus* infections. The family’s well water was reportedly treated. The patient’s only animal exposures were her two dogs at home, neither of which had any recent health issues or unusual skin lesions. The patient denied systemic symptoms, including fevers or chills. The patient had remained on clindamycin throughout development of the new skin lesions. She was also on prophylaxis with levofloxacin, acyclovir, pentamidine, and micafungin, as prescribed by her pediatric oncologist.

On admission, her skin lesions were initially presumed to be cellulitis and furunculosis caused by *Streptococcus* or *Staphylococcus* spp., so clindamycin was continued. Cefepime was also added for gram-negative coverage, including *Pseudomonas* sp., given her immunocompromised status. The skin lesion on her left hip was biopsied by a pediatric dermatologist, and bacterial and fungal cultures were obtained. The fungal culture began growing aseptate hyphae, concerning for mucormycosis, and the patient was started on isavuconazole, a newer broad-spectrum azole. A fungal blood culture was drawn, which demonstrated no growth. A whole-body computerized tomography scan and fundoscopic exam were performed to assess for invasive fungal disease, which did not reveal any other foci of disseminated fungal disease. Two days later, histopathology from the skin biopsy revealed broad, aseptate hyphae that were Gomori methenamine silver (GMS) stain positive. They were described as “sausage-like,” branching at 90° angles, and surrounded by dermal and subcutaneous acute and chronic inflammation with extensive necrosis. There were microabscesses in the specimen but no signs of angioinvasion. The specimens were then sent to the Fungus Testing Laboratory, University of Texas Health Science Center at San Antonio, for definitive species identification by DNA sequencing and antifungal susceptibility testing following the Clinical and Laboratory Standards Institution (CLSI) broth microdilution methods [8]. Amphotericin B was started for combination therapy for suspected mucormycosis as invasive mucormycosis carries significant morbidity and mortality. Pediatric surgery debrided the left hip lesion, and surgical pathology was consistent with the skin biopsy.

The patient’s gluteal lesions initially had a different appearance from the left hip lesion. Four days after surgical excision of the left hip lesion, the three gluteal lesions started draining fluid. The gluteal lesions appeared indurated, with purple discoloration, mild warmth and no tenderness to palpation. Fungal and bacterial cultures of the gluteal lesions were obtained at that time and demonstrated no growth. Without significant clinical improvement on clindamycin and cefepime, pediatric surgery excised these lesions, and dermatopathology revealed granulomatous inflammation with GMS positive fungal organisms in all three specimens.

**Fig. 1:**
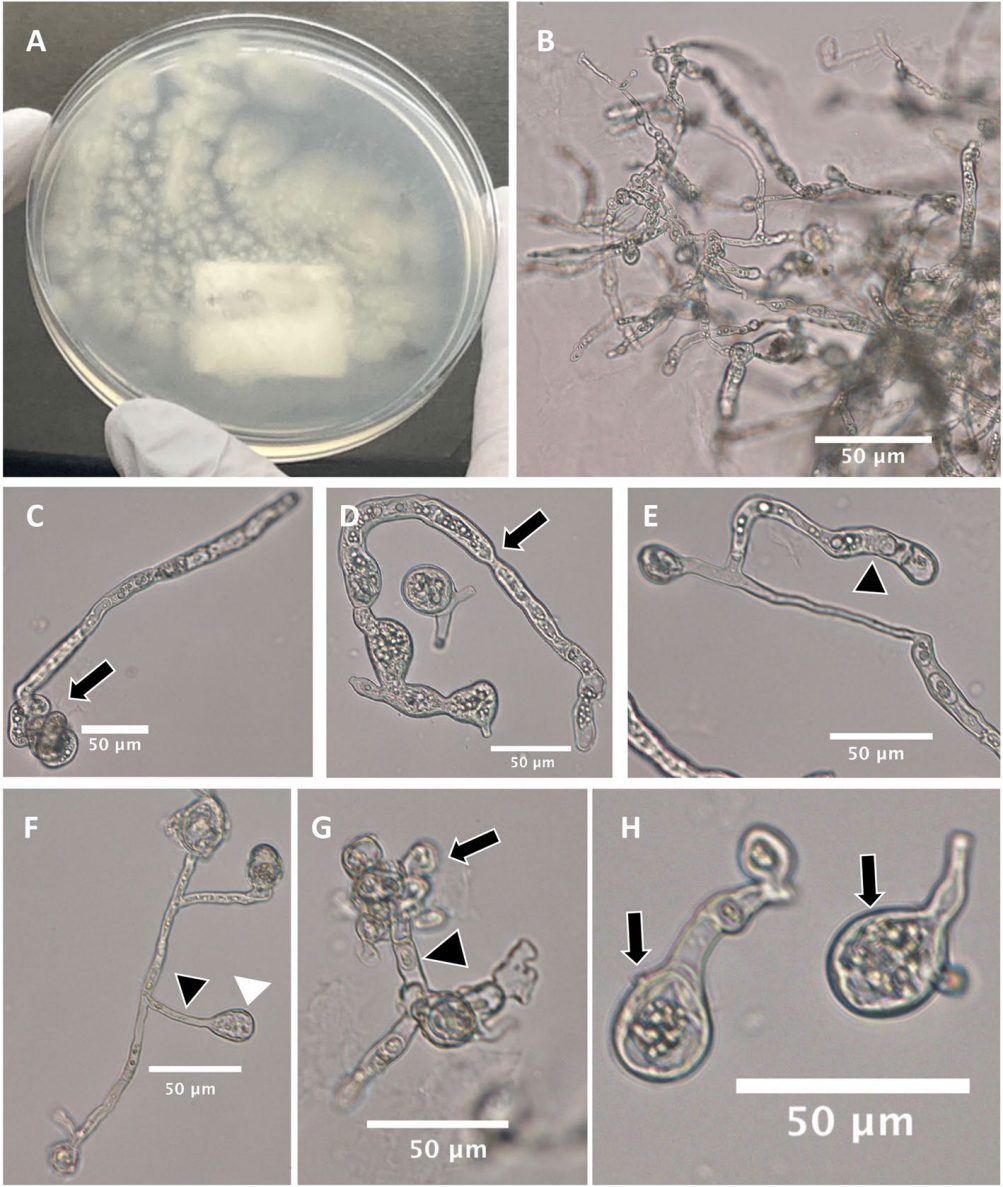
*Lagenidium deciduum* UTHSCSA DI23–100. **A** Cream glabrous colony on peptone yeast glucose agar plate. Microscopic characteristics from mounts taken from Saboraud’s dextrose broth culture at 25 C, 3 days. **B** Branching mycelioid structures. **C** Coiled hypha (arrow). **D** Vacuolated mycelioid structures constricted at septum resulting in the formation of segments (arrow). **E**, **F** Development of exit tubes (black arrowhead) arising laterally or terminally from thallus giving rise to vesicles at the tip (white arrowhead). **G** Zoospores (arrow) at tip of exit tube (arrowhead). **H** Encysted zoospores developing hypha (arrow)

**Fig. 2:**
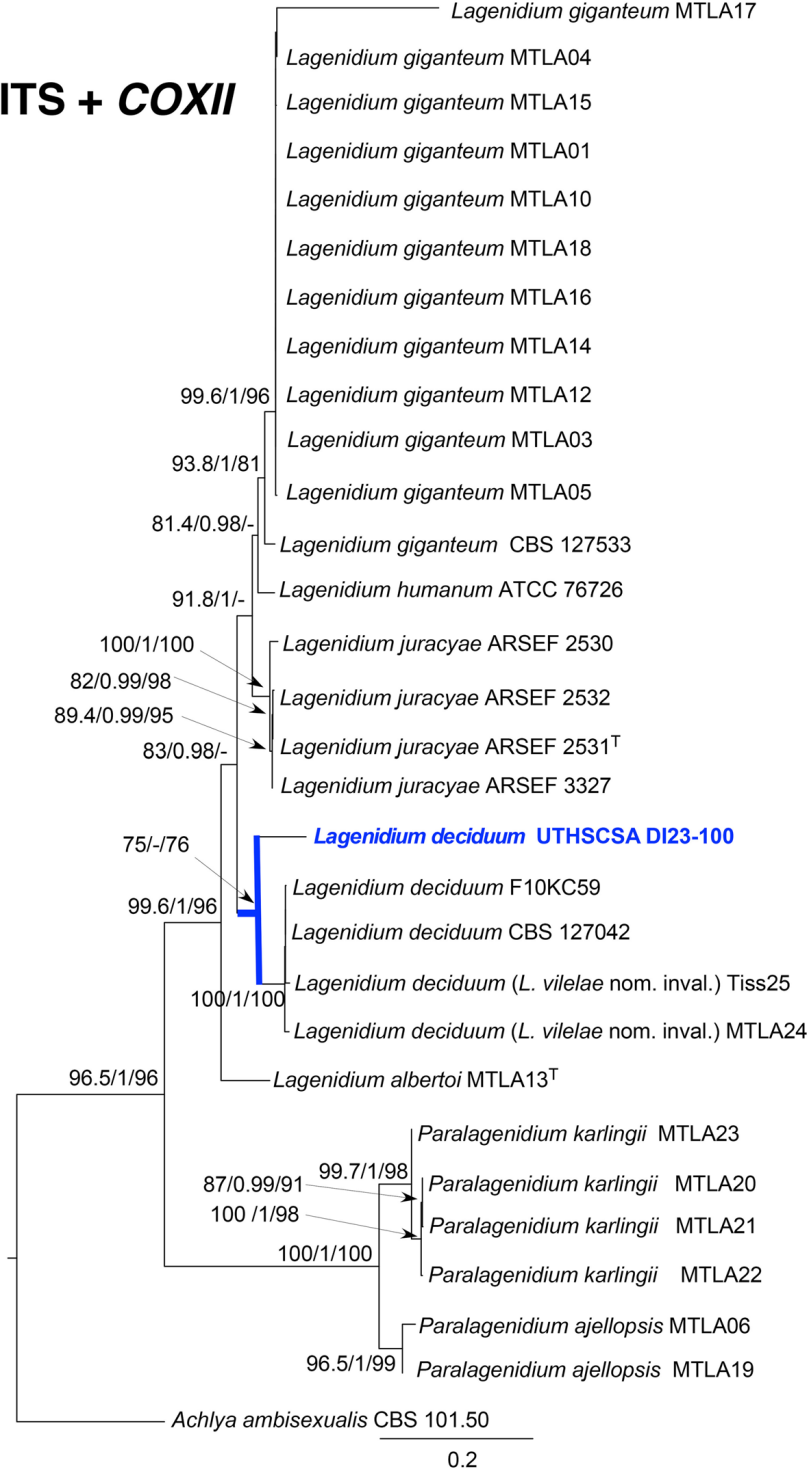
Phylogenetic tree

Genomic DNA was extracted from cultures grown on potato flakes agar. Phenotypic characterization, DNA sequencing and phylogenetic analysis of the internal transcribed spacer region of the nuclear rDNA (ITS) and the mitochondrial Cytochrome c oxidase subunit II (*COXII*) identified the causal organism as *Lagenidium deciduum*, as seen in Figs. 1 and 2 (GenBank accession numbers OR165095 and OR167557, respectively). Susceptibilities revealed the organism was not sensitive to amphotericin B, anidulafungin, caspofungin, fluconazole, itraconazole, micafungin, posaconazole, voriconazole, and isavuconazole (MIC/MEC values all greater than 8 μg/mL). However, when it comes to interpreting MIC data for fungi, clinical correlation cannot always be predicted. The patient’s antifungal regimen was adjusted from amphotericin B and isavuconazole to micafungin since oomycetes have abundant β-glucans in their cell walls [9]. Azithromycin, doxycycline, and terbinafine were also added because these antibiotics in combination have been effective in treating human pythiosis [10, 11].

Maximum likelihood tree based on combined ITS and *COXII* showing the relationship of isolate UTHSCSA DI23–100 with other *Lagenidium* species. Homologous sequences were aligned using Muscle and analyzed using IQ-TREE v.2.2.2.6 for the maximum likelihood method, SH-alrt, aBayes and Ultrafast bootstrap [12–15]. The combined data sets included sequences of taxa listed in Spies et al., 2016 and Vilela et al., 2019 [5, 16].

**Fig. 3:**
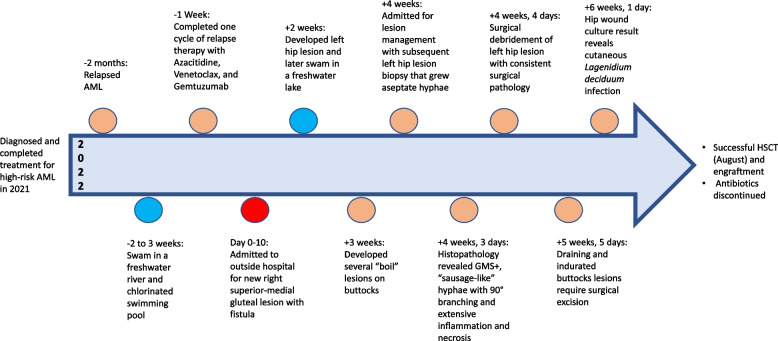
Timeline of clinical course

All excision sites healed well. She was discharged home on oral doxycycline, azithromycin, terbinafine, and IV micafungin. The patient enrolled in a clinical trial (NCT05322850) to receive an allogeneic stem cell transplant for her AML and was readmitted in August 2022 for HSCT. Antifungal treatment was continued through her bone marrow transplant until engraftment occurred. She was discharged home without recurrence of her skin lesions, and she has remained without evidence of fungal disease at more than 100 days post-transplant. Her full clinical course is outlined in Fig. 3.

## Discussion and conclusions

*Lagenidium deciduum* UTHSCSA DI23–100 formed a single clade in the combined ITS and *COXII* phylogenetic tree, separate from other clades representing other species of *Lagenidium* (Fig. 2). Although it grouped with the *L. deciduum* clade, this clade is weakly supported. Nevertheless, we refrain from describing this isolate as a novel species closely related to *L. deciduum* because this is only a single isolate and although it is phylogenetically distant, it is morphologically indistinguishable from *L. deciduum.* In this report, we presented the first case of a cutaneous *L. deciduum* infection in a human patient, and the first documented case of a *L. deciduum* infection in an immunocompromised host of any species.

Until now, the only other documented human case of *Lagenidium* infection had been an ocular infection that occurred in a patient in Thailand [17]. There was one other suspected case of a *Lagenidium* infection in a human patient that was published in an abstract in 2004 [18]. The patient presented with chronic cellulitis of the lower extremity, and initially the organism was identified as *Lagenidium* sp. However, subsequent analysis revealed the organism was actually *Paralagenidium karlingii*, which represented a new genus within the oomycete class.

Ultimately, there are no established guidelines for treating *Lagenidium* infections in humans because they are exceedingly rare. In an in vitro study, the growth of *Lagenidium* sp. was found to be reduced by caspofungin and terbinafine, although the magnitude of inhibition was only moderate [19]. In this same study, itraconazole, posaconazole, and voriconazole had limited activity against *Lagenidium*. Limited activity of azole antifungals is to be expected since oomycete cell membranes contain minimal ergosterol. On the other hand, susceptibility to caspofungin is plausible, as the cell wall of *Lagenidium* sp. contains abundant β-glucans, and caspofungin acts via inhibition of β (1,3)-d-glucan synthase. The mechanism of susceptibility to terbinafine is hypothesized to be from toxic intracellular accumulation of squalene [19].

In veterinary medicine, drug therapy has not been successful in treating *Lagenidium* infections. Early aggressive surgical resection with wide margins is the mainstay of treatment [9]. In a case series of six infected dogs, three of the dogs were treated with trials of antifungal and antibiotic therapy (including combinations of amphotericin B, itraconazole, terbinafine, enrofloxacin, cephalexin, amoxicillin-clavulanic acid, and lufenuron), none of which were successful [2]. Two of the dogs died due to great vessel rupture, and the other four were euthanized due to progressive disease. In a subsequent case report, a dog presented with a mandibular mass secondary to *Lagenidium giganteum* forma *caninum* infection*.* Despite surgical resection, itraconazole, prednisone and hyperbaric oxygen therapy, the mass invaded the calvaria and caused severe meningoencephalitis [20].

Based on similarity of presentation, treatment of lagenidiosis may be approached similarly to treatment of pythiosis in humans as both microorganisms are aquatic oomycetes typically found in subtropical and tropical regions. The mainstay of management for pythiosis is also surgical intervention, as the organism is often resistant to medical treatment [3]. For unresectable disease, prolonged combination treatment with itraconazole and terbinafine has been successful [21]. Adjunctive antibacterial agents have also successfully treated patients, including combinations of doxycycline with a macrolide and itraconazole or voriconazole [11].

This case illustrates a patient that was successfully treated with surgical excision, in accordance with veterinary lagenidiosis and human pythiosis management. Initially, there was concern for mucormycosis, and she was treated with isavuconazole and amphotericin B. Once the diagnosis of *L. deciduum* was established, she was transitioned to micafungin and terbinafine. The patient’s susceptibility testing was not helpful in guiding treatment, as the organism seemed to be resistant to every antifungal tested. The decision to transition her to micafungin and terbinafine was based off of in vitro studies on the susceptibility of *Lagenidium* sp. to various antifungals, as well as cases of successfully treated human pythiosis [19, 21]. Doxycycline and azithromycin were added to her treatment regimen based off of pythiosis treatment [11]. However, the patient did not continue on these antibiotics for as long of a duration as she took the antifungals due to worsening renal function. It is unclear whether the addition of these antibiotics is necessary in treating *Lagenidium* infections. Further in vitro studies should examine whether *Lagenidium* sp. are susceptible to macrolides and tetracyclines like *P. insidiosum.* Additionally, the patient was previously on micafungin prophylaxis when the skin lesions developed. It is possible that her skin lesions did not progress as quickly as cases of cutaneous pythiosis in neutropenic patients because of this. Alternatively, her skin lesions may not have progressed as quickly because she did not have severe neutropenia at the time or because *Lagenidium* sp. may not be as virulent as *P. insidiosum* in humans.

The optimal duration of antifungal therapy was also unknown for this patient. The patient was awaiting a bone marrow transplant to treat her relapsed AML. Given what is known about *Lagenidium* infections in canines, there was concern that any residual infection could progress or disseminate as she became severely neutropenic for a prolonged period of time following HSCT. After all her lesions were excised and healed, she was continued on antifungals, and it was deemed relatively safe to continue with her bone marrow transplant. The decision was made to continue antifungals until she achieved engraftment and thus reconstituted her immune system. This was consistent with the case of human pythiosis in a neutropenic Ewing sarcoma patient, where treatment was continued until the patient completed chemotherapy [6].

Regardless of the immune status of the patient, it is likely best practice to manage human *Lagenidium* infections with surgical excision when possible [3]. Whether immunocompetent hosts with pathogen-free surgical margins need to continue long-term antifungal therapy is unknown, but it may be advisable considering management recommendations in animals.

Ultimately, further research is needed to better understand the optimal treatment for this emerging pathogen, particularly in immunocompromised hosts. It is crucial to increase clinician awareness of human oomycosis. *Lagenidium* infections should be considered in the differential diagnosis of unusual cutaneous lesions that are resistant to typical antibacterial/antifungal treatment. The index of suspicion for oomycete infections should be even higher in immunocompromised patients and patients who have had exposure to freshwater lakes or ponds, especially in high-risk regions like the southern United States. Immunocompromised patients who live in these regions should consider avoiding these water sources, particularly since the spread of infection may be dramatically faster and harder to treat. Early detection of *Lagenidium* infections is of vital importance, as prompt surgical management with possible adjuvant medical therapy may prevent vascular invasion and dissemination of the disease to other organs.

## Data Availability

All data generated or analysed during this study are included in this article.

## Abbreviations

AML: Acute myeloid leukemia
HSCT: Hematopoietic stem cell transplant
GMS: Gomori methenamine silver
CLSI: Clinical and laboratory standards institute
ITS: Internal transcribed spacer region of nuclear rDNA
COXII: Cytochrome c oxidase subunit II
